# Stimulating effect of graphene oxide on myogenesis of C2C12 myoblasts on RGD peptide-decorated PLGA nanofiber matrices

**DOI:** 10.1186/s13036-015-0020-1

**Published:** 2015-11-25

**Authors:** Yong Cheol Shin, Jong Ho Lee, Min Jeong Kim, Suck Won Hong, Bongju Kim, Jung Keun Hyun, Yu Suk Choi, Jong-Chul Park, Dong-Wook Han

**Affiliations:** Department of Optics and Mechatronics Engineering, BK21+ Nano-Integrated Cogno-Mechatronics Engineering, College of Nanoscience & Nanotechnology, Pusan National University, Busandaehak-ro 63beon-gil, Geumjeong-gu, Busan, 609-735 >Korea; Clinical Dental Research Institute, Seoul National University Dental Hospital, Seoul, 03080 Korea; Department of Rehabilitation Medicine, College of Medicine, Cheonan, 330-714 Korea; Department of Nanobiomedical Science & BK21+ NBM Global Research Center, Cheonan, 330-714 Korea; Institute of Tissue Regeneration Engineering, Dankook University, Cheonan, 330-714 Korea; School of Anatomy, Physiology, and Human Biology, University of Western Australia, Crawley, WA 6009 Australia; Cellbiocontrol Laboratory, Department of Medical Engineering, Yonsei University College of Medicine, 50-1, Yonsei-ro, Seodaemun-gu, Seoul, 120-752 Korea

**Keywords:** Graphene oxide, RGD peptide, Poly(lactic-*co*-glycolic acid), Nanofiber matrix, Myogenic differentiation

## Abstract

**Background:**

In the field of biomedical engineering, many studies have focused on the possible applications of graphene and related nanomaterials due to their potential for use as scaffolds, coating materials and delivery carriers. On the other hand, electrospun nanofiber matrices composed of diverse biocompatible polymers have attracted tremendous attention for tissue engineering and regenerative medicine. However, their combination is intriguing and still challenging.

**Results:**

In the present study, we fabricated nanofiber matrices composed of M13 bacteriophage with RGD peptide displayed on its surface (RGD-M13 phage) and poly(lactic-*co*-glycolic acid, PLGA) and characterized their physicochemical properties. In addition, the effect of graphene oxide (GO) on the cellular behaviors of C2C12 myoblasts, which were cultured on PLGA decorated with RGD-M13 phage (RGD/PLGA) nanofiber matrices, was investigated. Our results revealed that the RGD/PLGA nanofiber matrices have suitable physicochemical properties as a tissue engineering scaffold and the growth of C2C12 myoblasts were significantly enhanced on the matrices. Moreover, the myogenic differentiation of C2C12 myoblasts was substantially stimulated when they were cultured on the RGD/PLGA matrices in the presence of GO.

**Conclusion:**

In conclusion, these findings propose that the combination of RGD/PLGA nanofiber matrices and GO can be used as a promising strategy for skeletal tissue engineering and regeneration.

## Background

The extracellular matrix (ECM) is a network structure composed of various biomolecules, such as collagen, elastin, fibrous protein and cell adhesion protein. The ECM controls the cellular behaviors and provides a favorable microenvironment for the growth of cells [1–3]. In recent years, there have been vigorous efforts to develop three-dimensional scaffolds with not only a suitable structure, but also biofunction for supporting cellular behaviors [4–6]. The three-dimensional scaffolds can be fabricated using various techniques such as air-spinning, phase separation, emulsion templating, and salt leaching [7, 8]. Among the techniques, electrospinning is an effective and suitable technique for fabricating three-dimensional scaffolds because electrospun matrices have similar structure to the natural ECM, large surface area-to-volume ratio and high porosity [8]. Consequently, electrospun matrices composed of various biomaterials, such as collagen, polyurethane, poly(hydroxybutyrate), and poly(lactic-*co*-glycolic acid, PLGA), have been applied as tissue engineering scaffolds [9, 10]. Among the various biomaterials used for tissue engineering scaffolds, RGD peptide is one of the most well-known peptides for enhancing the cellular behaviors [11, 12]. The RGD peptide is a tripeptide (Arg-Gly-Asp) found on ECM proteins and a major recognition sequence for integrin that plays an important role in cell adhesion. Therefore, RGD peptide-functionalized substrates can enhance the cellular behavior. In particular, RGD peptide-conjugated substrates based on various biocompatible polymers, such as chitosan, polycaprolactone, poly (_L_-lactide), polystyrene, and PLGA, have been developed as tissue engineering scaffolds [13–16].

Graphene, a single layer of the sp^2^ carbon network with a honeycomb-lattice structure, and its derivatives are considered attractive candidates for biomedical applications including biosensors, scaffolds for tissue engineering, and substrates for the differentiation of stem cells [17–20]. In particular, previous reports have shown that graphene oxide (GO) is non-cytotoxic and has the ability to stimulate the differentiation of myoblasts [21–23]. Therefore, GO can be used to stimulate and promote myogenic differentiation for skeletal tissue regeneration.

Until now, electrospun matrices composed of a wide variety of biodegradable copolymers have been extensively developed for tissue engineering and regeneration as well as drug and gene delivery [9, 24, 25]; however, their use in combination with GO is novel. In the present study, nanofiber matrices composed of PLGA and M13 bacteriophage with RGD peptide displayed on its surface (RGD-M13 phage) were prepared via electrospinning. PLGA is a biodegradable polymer with good cell compatibility that is widely used for the fabrication of tissue engineering scaffolds [26–28]. The M13 phage is a bacterial virus with a long-rod structure [29]. The M13 phage does not have harmful effects on mammalian and human cells [30]. On the other hand, the surface of M13 phage is covered by 2700 major coat proteins (pVIII) that can be modified to express many desired peptides through genetic engineering [31, 32]. In addition, it is conveniently produced by infecting bacterial cell and mass amplification process [33]. Therefore, RGD peptide decoration by using M13 phage is very efficient. The PLGA decorated with RGD-M13 phage (RGD/PLGA) nanofiber matrices were characterized by atomic force microscopy (AFM), Fourier-transform infrared (FTIR) spectroscopy, Raman spectroscopy and thermogravimetric analysis (TGA). Moreover, the cellular behaviors of C2C12 mouse myoblasts on the RGD/PLGA nanofiber matrices were evaluated when GO was added in the culture media to determine if a combination of GO and matrices can be applied as a novel strategy for skeletal tissue regeneration.

## Results and discussion

### Characterizations of RGD/PLGA nanofiber matrices

**Fig. 1:**
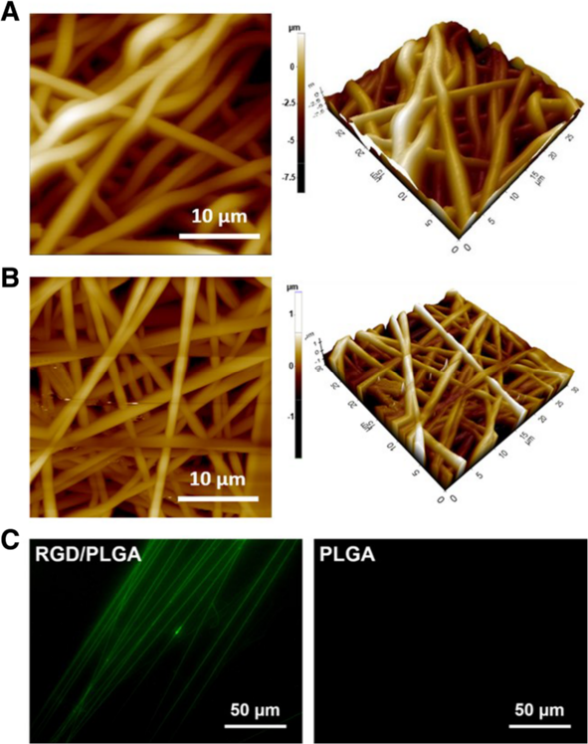
Surface morphology and immunostaining of electrospun nanofibers. AFM images of (**a**) PLGA and (**b**) RGD/PLGA nanofiber matrices. **c** Immunofluorescence images of PLGA and RGD/PLGA electrospun fibers. RGD peptide-displaying M13 phages in the RGD/PLGA nanofibers were immunostained with the FITC-labeled anti-M13 phage antibody (green). All photographs shown in this figure are representative of six independent experiments with similar results

The PLGA and RGD/PLGA nanofiber matrices were fabricated by electrospinning and are shown in Fig. 1a and b. They had a continuous and three-dimensional network structure with interconnected pores that were similar to the natural ECM. The average diameters of the PLGA and RGD/PLGA electrospun fibers were 1440 ± 150 and 200 ± 30 nm, respectively. The diameter of the fabricated nanofibers decreased when RGD-M13 phages were blended (Fig. 1a and b). It was reported that the diameter of the electrospun nanofibers was dependent on the properties of the electrospinning solution, such as viscosity, electrical conductivity, chemical composition, and molecular weight of the constituent polymers [34–36]. When RGD-M13 phage suspension was blended with PLGA, the electrical conductivity of the electrospinning solution was increased due to the salts of the RGD-M13 phage suspension. In addition, the viscosity of the electrospinning solution decreased with the blending of the RGD-M13 phage suspension. Therefore, the diameter of the RGD/PLGA nanofiber decreased compared to that of the PLGA fiber [34]. A decrease in the diameter of the RGD/PLGA nanofibers leads to a significant increase in the surface area-to-volume ratio of the matrices, which in turn leads to an enhanced interaction between the cells and RGD/PLGA matrices [37–39]. Immunofluorescence staining of the RGD-M13 phage was conducted to confirm the presence of the RGD-M13 phage in the hybrid nanofibers. As shown in Fig. 1c, the green fluorescence of the RGD-M13 phages was detected along the RGD/PLGA nanofibers. On the other hand, no fluorescence was detected in the PLGA fiber. It was revealed that the RGD-M13 phages were distributed along the RGD/PLGA nanofibers.

**Fig. 2:**
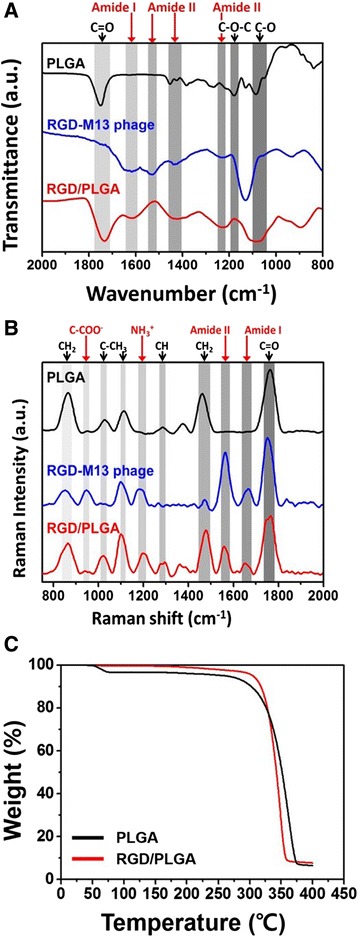
Characterization of the PLGA and RGD/PLGA nanofiber matrices. **a** FTIR and (**b**) Raman spectra of the PLGA matrices, RGD-M13 phages and RGD/PLGA nanofiber matrices. **c** Thermogravimetric analysis (TGA) curves of the PLGA and RGD/PLGA nanofiber matrices

FTIR and Raman spectroscopy were conducted to analyze the composition of the matrices. Figure 2a shows the FTIR spectra of the PLGA matrices, RGD-M13 phages and RGD/PLGA matrices. The typical bands of PLGA were observed at 1750 and 1080 cm^−1^, which represented the C = O stretching and C-O stretching modes of the ester group, respectively [40]. Additionally, another band was observed at 1180 cm^−1^, which was assigned to the C-O-C stretching [41]. On the other hand, in the RGD/PLGA matrices, the characteristic bands of RGD-M13 phage were found near 1630, 1450 and 1280 cm^−1^, which can be attributed to the amide I, II and III vibrations from protein structure, respectively [42, 43]. This result was well supported by Raman spectrum of RGD/PLGA matrices which showed the characteristic peaks of RGD-M13 phages. As shown in the Fig. 2b, the peaks for the RGD-M13 phages were apparently observed near 1200, 1560 and 1660 cm^−1^, which can be assigned to the NH_3_^+^, amide II and amide I groups, respectively [44–46]. The thermal stability of the matrices was evaluated by TGA (Fig. 2c). The onset of the decomposition temperatures of the PLGA and RGD/PLGA nanofiber matrices were approximately 280 and 300 °C, respectively. No significant difference was observed between the two types of matrices. The results from the FTIR spectra and TGA revealed that the RGD/PLGA nanofibers were successfully fabricated and the RGD-M13 phages were evenly distributed throughout the matrices. Moreover, the addition of RGD-M13 phage did not adversely affect the thermal stability of the matrices, and the matrices were thermally stable under cell culture conditions. Therefore, it is suggested that the RGD/PLGA nanofiber matrices have a suitable structure and physicochemical properties as a cellular scaffold.

### Cytotoxicity of GO and growth of C2C12 myoblasts on RGD/PLGA nanofiber matrices

**Fig. 3:**
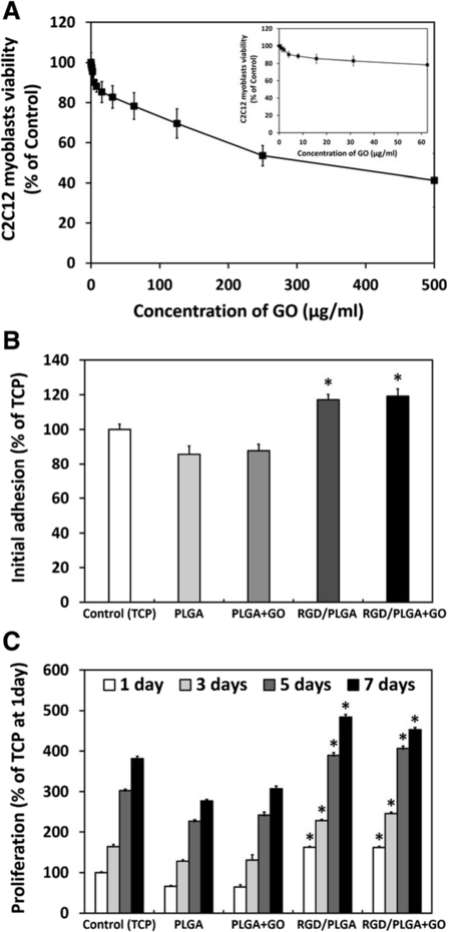
*In vitro* assays for cytotoxicity of GO and growth of C2C12 myoblasts on RGD/PLGA matrices. **a** C2C12 myoblast viability after the 24 h of incubation with GO. **b** Initial adhesion and (**c**) proliferation of C2C12 myoblasts cultured on the control (TCP), PLGA matrices and RGD/PLGA nanofiber matrices with or without the GO. The viability, initial adhesion and proliferation of C2C12 myoblasts were determined using a CCK-8 assay. An asterisk (*) denotes a significant difference between the control and other groups, *p* < 0.05. The data is expressed as mean ± SD based on at least duplicate observations from three independent experiments

The influence of GO on the growth of cells is highly dependent on its size, shape and concentration [47–49]. Therefore, to evaluate the cell compatibility of the GO and RGD/PLGA matrices, *in vitro* assays for cytotoxicity of GO and growth of C2C12 myoblasts on RGD/PLGA nanofiber matrices with 10 μg/ml of GO were conducted using a cell counting kit-8 (CCK-8) assay, based on the mitochondrial activity (Fig. 3). As shown in Fig. 3a, the viability of the C2C12 myoblasts decreased with increasing GO concentration. The cell viability was slightly (*p* < 0.05) decreased at concentrations lower than 62.5 μg/ml, whereas it was significantly (*p* < 0.05) decreased at higher concentrations (≥ 100 μg/ml). The C2C12 myoblasts viability decreased to approximately 40 % of the control at 500 μg/ml GO. According to previous literature, the cytotoxicity of GO involves the oxidative stress response [48]. GO has been reported to exhibit dose-dependent cytotoxicity but did not show apparent cytotoxicity at a low concentration [21, 48, 49]. Our results are in accordance with previous studies. In the present study, GO was cytotoxic to C2C12 myoblasts at high concentrations (≥ 100 μg/ml) but not at low concentrations (< 62.5 μg/ml) (Fig. 3a). These results indicated that the GO has no harmful effects on C2C12 myoblasts and is non-cytotoxic at 10 μg/ml.

The initial adhesion and proliferation of C2C12 myoblasts on the tissue culture plastics (TCPs), PLGA matrices, and RGD/PLGA nanofiber matrices were also evaluated. In addition, C2C12 myoblasts on the PLGA and RGD/PLGA matrices were incubated in the culture media containing 10 μg/ml of GO to examine the effects of GO. The initial adhesion of C2C12 myoblasts was significantly (*p* < 0.05) promoted on the RGD/PLGA matrices compared to that on the TCPs and PLGA matrices, regardless of the addition of GO (Fig. 3b). Moreover, as shown in Fig. 3c, the proliferation of C2C12 myoblasts was significantly (*p* < 0.05) increased on the RGD/PLGA matrices, while it was slightly done in the GO-added groups. RGD peptide-conjugated substrates were reported to enhance not only cell adhesion, but also proliferation [13–16]. In addition, these enhanced initial adhesion and proliferation might be also due to the fact that the increased surface area-to-volume ratio can facilitate the interaction between cells and matrices. On the other hand, the proliferation of C2C12 myoblasts was highest when they were cultured on the RGD/PLGA matrices supplemented with GO for 5 days of culture. According to previous reports, GO can also support cellular behaviors, including cell adhesion and proliferation [18, 19, 50]. At 7 days after culture, however, the increase in proliferation rate was reduced. This might be due to the fact that C2C12 myoblasts were sufficiently grown and began to differentiate into myotubes rather than proliferate [51, 52].

**Fig. 4:**
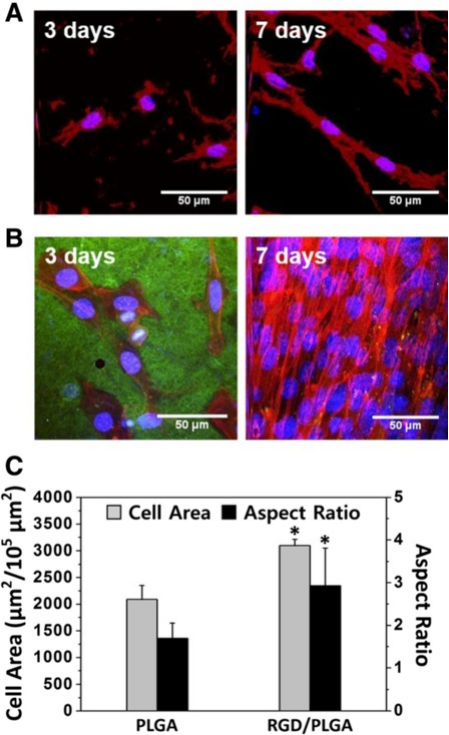
The morphologies of the C2C12 myoblasts on the PLGA and RGD/PLGA nanofiber matrices. Two-photon excitation fluorescence images of C2C12 myoblasts cultured on **a** PLGA and (**b**) RGD/PLGA nanofiber matrices for 3 and 7 days. The cell nuclei were counterstained by DAPI (blue), F-actins were stained by TRITC-labelled phalloidin (red) and RGD-M13 phages in the RGD/PLGA nanofiber matrices were immunostained with the FITC-labelled anti-M13 phage antibody (green). **c** Quantification of the cell area and aspect ratio at 3 days. The asterisk (*) denotes a significant difference between the PLGA and RGD/PLGA nanofiber matrices, *p* < 0.05. Quantitative analysis was performed using ImageJ Software

Figure 4 shows the morphologies of the C2C12 myoblasts on the PLGA and RGD/PLGA matrices for 3 and 7 days. As shown in Fig. 4a, the C2C12 myoblasts on the PLGA matrices were unable to form F-actin properly and the number of cells did not appreciably increase. On the other hand, the C2C12 myoblasts on the RGD/PLGA matrices showed a spindle-like morphology with well-organized F-actins and the number of cells was significantly increased (Fig. 4b). This can be explained by the fact that the RGD peptide moieties, which exist on the RGD/PLGA matrices, stimulate cell adhesion, and promote the proliferation of cells. Additionally, the green fluorescence of the RGD-M13 phages was observed throughout the RGD/PLGA nanofibers. These stimulated adhesion and proliferation of myoblasts were also demonstrated by quantitative analysis of the cell morphology (Fig. 4c). The cell area of the C2C12 myoblasts on the RGD/PLGA matrices were significantly (*p* < 0.05) increased by enhancing the adhesion (Fig. 4c). In addition, the aspect ratio of C2C12 myoblasts on the RGD/PLGA matrices was increased. Generally, the cellular extension of C2C12 myoblasts was favorable for the fusion of the neighboring cells and was considered to be the initiation of myogenic differentiation [23, 53, 54]. These results indicated that the RGD/PLGA nanofiber matrices effectively support the growth and differentiation of C2C12 myoblasts.

### Myogenic differentiation of C2C12 myoblasts on RGD/PLGA nanofiber matrices

**Fig. 5:**
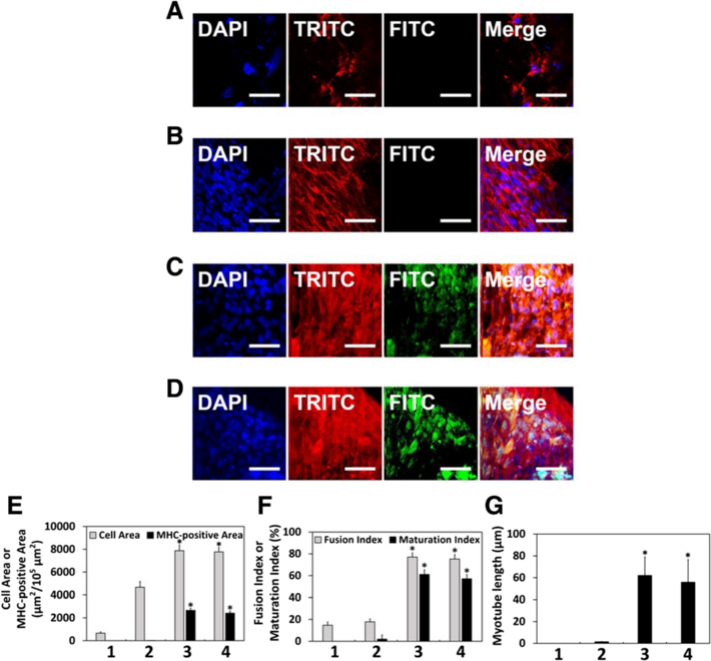
Stimulated myogenesis of C2C12 myoblasts on RGD/PLGA nanofiber matrices. Two-photon excitation fluorescence images of the C2C12 myoblasts on (**a**) PLGA and (**b**) RGD/PLGA nanofiber matrices without the GO, (**c**) RGD/PLGA nanofiber matrices with the GO and (**d**) RGD/PLGA nanofiber matrices in differentiation media (DM). The cell nuclei were counterstained by DAPI (blue), F-actins were stained with TRITC-labelled phalloidin (red) and the myosin heavy chains (MHCs) were stained with FITC-labelled anti-MHC antibody (green). The scale bars are 50 μm. All photographs shown in this figure are representative of six independent experiments with similar results. Quantification of (**e**) the cell area, MHC-positive area, (**f**) fusion index, maturation index, and (**g**) myotube length (1: PLGA matrices, 2: RGD/PLGA nanofiber matrices without the GO, 3: RGD/PLGA nanofiber matrices with the GO, and 4: RGD/PLGA nanofiber matrices in DM). An asterisk (*) denotes a significant difference between PLGA matrices and other groups, *p* < 0.05. Quantitative analysis was performed using ImageJ Software

The myogenic differentiation of C2C12 myoblasts was evaluated by immunofluorescence staining for myosin heavy chain (MHC). MHC is a well-characterized marker for the myogenic differentiation of skeletal muscle cells [55]. The C2C12 myoblasts were cultured on the PLGA or RGD/PLGA matrices and incubated in growth media (GM) containing 10 μg/ml of GO for 5 days. As shown in Fig. 5a, the C2C12 myoblasts on PLGA matrices showed an unusual morphology with poorly-developed F-actin network and cellular debris. In addition, they did not differentiate because the number of cells was insufficient to differentiate. It is known that the C2C12 myoblasts can be differentiated when they are fully proliferated in differentiation media (DM) [56, 57]. Therefore, the C2C12 myoblasts on the PLGA matrices could not be differentiated and the green fluorescence of MHC was not exhibited. The C2C12 myoblasts cultured on the RGD/PLGA matrices without the GO were well-grown but did not differentiate into mature myotubes, which is evidence of myogenic differentiation (Fig. 5b). On the RGD/PLGA matrices with the GO, however, the C2C12 myoblasts were fully proliferated and differentiated with multinucleate myotubes (Fig. 5c). In addition, the green fluorescence of MHC was observed from the myotubes. On the other hand, the C2C12 myoblasts cultured on the RGD/PLGA matrices in DM were differentiated as a positive control (Fig. 5d). This was also supported by quantification of the MHC-positive area, fusion index, maturation index, and myotube length. As shown in Fig. 5e, the cell area and MHC-positive area of the C2C12 myoblasts cultured on the RGD/PLGA matrices in the presence of the GO were significantly (*p* < 0.05) higher than those on PLGA and RGD/PLGA matrices in the absence the GO. No difference in cell area and MHC-positive area was observed between the GO-added groups and positive controls (cultured on RGD/PLGA matrices in DM). We also quantified the myogenic differentiation of C2C12 myoblasts by analyzing the fusion index, maturation index and myotube length. As shown in Fig. 5f and g, the fusion index, maturation index and myotube length significantly (*p* < 0.05) increased when the C2C12 myoblasts were cultured on the RGD/PLGA matrices in combination with GO or in DM. Consequently, it was revealed that the differentiation of the C2C12 myoblasts was effectively stimulated by the synergistic effect of the RGD/PLGA matrices and GO. The RGD/PLGA matrices promoted cell proliferation and GO stimulated cell differentiation. According to previous studies, the enhancement of cell adhesion and proliferation accelerates the differentiation of the C2C12 myoblasts [58–61]. In addition, many studies showed that GO can stimulate the differentiation of various types of cells as well as myoblasts [19, 20, 23, 62, 63]. Therefore, it is suggested that the RGD/PLGA matrices are suitable scaffolds for the growth of C2C12 myoblasts. Furthermore, their use in combination with GO can stimulate and accelerate the myogenic differentiation of C2C12 myoblasts.

## Conclusions

This study developed biofunctional scaffolds that can promote the proliferation and differentiation of C2C12 myoblasts. The RGD/PLGA nanofiber matrices were successfully fabricated by electrospinning and the characteristics of the matrices were investigated. Our results showed that the structure of the RGD/PLGA nanofiber matrices was dimensionally similar to that of the natural ECM and the RGD-M13 phages were homogeneously distributed in the matrices. It was confirmed that the RGD/PLGA matrices were cell compatible and could support the growth and proliferation of C2C12 myoblasts. In addition, when the matrices were used with GO, the myogenic differentiation of C2C12 myoblasts was effectively stimulated and accelerated. In conclusion, it is suggested that the RGD/PLGA nanofiber matrices are suitable scaffolds with the ability to support cellular behaviors. Moreover, the matrices can be used in combination with GO as a novel strategy for skeletal tissue regeneration and treatment of muscle dysfunction by stimulating the differentiation of myoblasts. Further studies with GO-loaded RGD/PLGA nanofiber matrices are needed to employ the approach presented in this study for *in vivo* applications.

## Methods

### Preparation of RGD/PLGA nanofiber matrices

RGD peptides were displayed on major coat protein that wraps the side wall of the M13 phage cloning method according to previously described procedure [31, 32]. Briefly, to incorporate the RGD peptide sequences, polymerase chain reaction was carried out using Phusion DNA Polymerase, two primers and an M13KE vector [64]. The engineered M13KE phages were amplified in bacterial cells and concentrated by polyethylene glycol precipitation.

The RGD/PLGA nanofiber matrices were prepared by electrospinning. Briefly, PLGA resins [PLA/PGA = 75/25, molecular weight = 70–110 kDa, Evonik Industries, Essen, Germany] were dissolved in 1, 1, 1, 3, 3, 3-hexafluoroisopropanol (HFIP, Sigma-Aldrich Co., St Louis, MO) at a concentration of 200 mg/ml. The RGD-M13 phage suspensions in tris buffered saline (TBS) buffer (50 mM tris and 150 mM NaCl, pH 7.4, Bioworld, Dublin, OH) were blended with the PLGA solution. The concentration of RGD-M13 phages was 10 mg/ml. The mixture solution of PLGA and RGD-M13 phages was loaded into a syringe fitted with a 25 G needle. A voltage of 14 kV was applied and the working distance between the needle tip and the collector was 11 cm. The flow rate of the mixture solution was 0.2 ml/h. Randomly-oriented RGD/PLGA nanofibers were collected on a steel rotating wheel covered with aluminum foil. The RGD/PLGA nanofiber matrices were then dried overnight under vacuum at room temperature (RT) to remove any residual solvent.

### Characterizations of RGD/PLGA nanofiber matrices

The surface morphology of the RGD/PLGA nanofiber matrices was observed by AFM (NX10, Park Systems Co., Suwon, Korea) with a Multi 75 silicon scanning probe. Immunofluorescence staining for RGD-M13 phages was conducted to examine the existence of RGD-M13 phages in the RGD/PLGA nanofibers. The RGD/PLGA nanofibers were incubated with the primary anti-M13 phage antibody for 2 h at RT and then incubated with secondary fluorescein isothiocyanate (FITC)-conjugated goat anti-rabbit IgG for 1 h at RT. The immunostained nanofibers were imaged with an Olympus IX81 inverted fluorescence microscope. The composition of the PLGA, RGD-M13 phage, and RGD/PLGA nanofiber matrices was characterized by FTIR and Raman spectroscopy. The FTIR spectra were collected using a FTIR spectrophotometer (Nicolet 560, Nicolet Co., Madison, WI). All spectra were recorded in absorption mode in the wavelength range of 800–2000 cm^−1^ with a resolution of 4.0 cm^−1^ and 16-times scanning. The Raman spectra were obtained by a Raman spectroscope (Micro Raman PL Mapping System, Dongwoo Optron Co., Ltd, Gwangju-si, Korea) with an Ar-ion laser of wavelength 514.5 nm at a power of 5 mW. The FTIR and Raman spectra were baselined to minimize the effect of the background (slope) by using ORIGIN 8.0^®^ program (OriginLab Corporation, Northampton, MA). The thermal stability of the PLGA and RGD/PLGA nanofiber matrices was evaluated by TGA (TGA n-1000, Scinco Co., Seoul, Korea). Samples were weighed (approximately 5 mg) in open aluminum pans and heated from 25 to 400 °C at a heating rate of 10 °C/min.

### Cytotoxicity of GO and growth of C2C12 myoblasts

The C2C12 mouse myoblasts were purchased from the American Type Culture Collection (Rockville, MD) and routinely maintained in GM, Dulbecco’s modified Eagle’s Medium (DMEM, Welgene, Daegu, Korea) supplemented with 10 % fetal bovine serum (FBS, Welgene) and a 1 % antibiotic-antimycotic solution (containing 10,000 units penicillin, 10 mg streptomycin and 25 μg amphotericin B per ml, Sigma-Aldrich Co.) at 37 °C in a humidified atmosphere containing 5 % CO_2_.

GO was prepared from expanded graphite according to the modified Hummers and Offeman method as described in our previous work [65, 66]. The cytotoxicity of GO for the C2C12 myoblasts was assessed using a CCK-8 assay (Dojindo, Kumamoto, Japan) according to the manufacturer’s instructions. The number of viable cells was found to be directly proportional to the metabolic reaction products obtained in the CCK-8 assay [24, 67]. Briefly, the C2C12 myoblasts were seeded at a density of 5 × 10^4^ cells/ml on 24-well plates in 1 ml of GM and incubated for 24 h. Subsequently, GO was added with increasing concentrations (0 to 500 μg/ml) to the culture media and the cells were incubated with a CCK-8 solution for the last 2 h of the culture period (24 h) at 37 °C in the dark. The absorbance was measured at 450 nm using an ELISA reader (SpectraMax 340, Molecular Device Co., Sunnyvale, CA).

The initial adhesion and proliferation of C2C12 myoblasts was determined by a CCK-8 assay, as described above. The cells were seeded on the PLGA and RGD/PLGA matrices at a density of 1 × 10^4^ cells/ml in 1 ml of GM. To examine the effects of GO, the C2C12 myoblasts on the PLGA and RGD/PLGA matrices were cultured in GM containing 10 μg/ml of GO. Each cell culture was incubated with a CCK-8 solution for the last 2 h of the culture period for initial adhesion (6 h) and proliferation (1, 3, 5 and 7 days) at 37 °C in the dark. Parallel sets of C2C12 myoblasts were cultured on TCPs and results were regarded as positive (+) controls.

### Immunofluorescence staining of C2C12 myoblasts

For the morphological observations, C2C12 myoblasts cultured on the PLGA or RGD/PLGA matrices for 3 and 7 days were fixed with a 3.7 % formaldehyde solution (Sigma-Aldrich Co.) for 10 min. After fixation, the cells were immersed in a 0.1 % Triton X-100 (Sigma-Aldrich Co.) solution for 5 min and then blocked with a 2 % bovine serum albumin (BSA, GenDEPOT, Barker, TX) solution in Dulbecco’s phosphate-buffered saline (DPBS, Gibco BRL, Rockville, MD) for 30 min. After blocking, the cells were incubated with TRITC-labeled phalloidin (Molecular Probes, Eugene, OR) for 20 min at RT. The cell nuclei were counterstained with 1 μM of 4',6-diamidino-2-phenylindole (DAPI, Sigma-Aldrich Co.). The C2C12 myoblasts-cultured matrices were immunostained with the primary and secondary antibodies as described in ‘Characterizations of RGD/PLGA nanofiber matrices’ section of Methods. The fluorescence images were observed using a custom-built two-photon excitation fluorescence microscope, as described elsewhere [68, 69]. The fluorescence images were analyzed using ImageJ software (National Institutes of Health, Bethesda, MD). Quantitative analysis of the cell morphology was carried out by measuring the cell area and calculating the aspect ratio.

To examine the myogenic differentiation of C2C12 myoblasts, the MHCs were stained with the Alexa Fluor 488 conjugated anti-MHC monoclonal antibody (eBioscienceInc., San Diego, CA). The C2C12 myoblasts were cultured on the PLGA or RGD/PLGA matrices for 24 h. The cells on the matrices were then incubated in GM or DM containing 10 μg/ml of GO. The fluorescence images were observed after the additional 5 days of incubation. The C2C12 myoblasts were cultured on the RGD/PLGA matrices in DM (low-serum media, DMEM containing 2 % of horse serum and 1 % antibiotic-antimycotic solution) and the results were regarded as positive (+) controls. Quantitative analysis of the myogenic differentiation of C2C12 myoblasts was conducted by analyzing the fusion index, maturation index and myotube length. The fusion index was obtained by calculating the percentage of the number of nuclei within the multinucleate myotubes containing more than two nuclei to the total number of nuclei. The maturation index was calculated as the percentage of myotubes containing more than five nuclei to the total number of myotubes [23].

### Statistical analysis

All variables were tested in three independent cultures for each experiment, which was repeated twice (n = 6). All the quantitative data is expressed as the mean ± standard deviation (SD). Statistical comparisons were carried out by a one-way analysis of variance (ANOVA), followed by a Bonferroni test for multiple comparisons. A value of *p* < 0.05 was considered statistically significant.

## Abbreviations

AFM: Atomic force microscopy
ANOVA: Analysis of variance
BSA: Bovine serum albumin
CCK-8: Cell counting kit-8
DAPI: 4',6-Diamidino-2-phenylindole
DM: Differentiation media
DMEM: Dulbecco’s modified Eagle’s Medium
DPBS: Dulbecco’s phosphate-buffered saline
ECM: Extracellular matrix
FBS: Fetal bovine serum
FITC: Fluorescein isothiocyanate
FTIR: Fourier-transform infrared
GM: Growth media
GO: Graphene oxide
HFIP: 1, 1, 1, 3, 3, 3-Hexafluoroisopropanol
MHC: Myosin heavy chain
PLGA: Poly(lactic-*co*-glycolic acid)
RGD peptide: Arg-Gly-Asp
RGD/PLGA: PLGA decorated with RGD-M13 phage
RGD-M13 phage: M13 bacteriophage with RGD peptide displayed on its surface
RT: Room temperature
SD: Standard deviation
TBS: Tris buffered saline
TCP: Tissue culture plastic
TGA: Thermogravimetric analysis
